# How and Where *Periglandula* Fungus Interacts with Different Parts of *Ipomoea asarifolia*

**DOI:** 10.3390/jof8080823

**Published:** 2022-08-06

**Authors:** Yanisa Olaranont, Alyssa B. Stewart, Wisuwat Songnuan, Paweena Traiperm

**Affiliations:** Department of Plant Science, Faculty of Science, Mahidol University, Rama VI Road, Bangkok 10400, Thailand

**Keywords:** Clavicipitaceae, ergoline alkaloids, morning glory, plant–fungal symbiosis, secondary metabolites

## Abstract

*Periglandula* is a fungal genus that is associated with plants in the family Convolvulaceae. They produce medicinally important constituents called ergot alkaloids, which are stored in their host plants. Previously, the fungi were reported to mainly interact with young leaves and seeds of Convolvulaceae species. However, knowledge about how ergot alkaloid-producing fungi interact with their host plants is still lacking. Therefore, we investigated the interaction of *Periglandula* fungus with different plant parts of *Ipomoea asarifolia,* using molecular, histochemical, anatomical and micromorphological techniques. Our findings confirm the presence of *Periglandula ipomoeae* on six out of the eight plant parts examined (young folded leaves, mature leaves, flower buds, mature flowers, young seeds and mature seeds). The fungus was mostly distributed along external plant surfaces, and particularly on areas that were relatively unexposed. Our results suggest that the density of fungal mycelium varies depending on glandular trichome density and the growth stage of the host plant. Detection of the fungus in the flowers of its host plant, for the first time, fills a missing link in understanding how vertical transmission of *Periglandula* species occurs.

## 1. Introduction

*Periglandula* is a recently discovered fungal genus that belongs to the Clavicipitaceae in Hypocreales [1]. The family Clavicipitaceae is known to include epibiotic, endophytic, and parasitic fungi associated with monocotyledonous plants in the families Cyperaceae, Poaceae, and Juncaceae [2,3,4]. One distinguishable character of the fungi in this family is their role in ergoline (syn. ergot) alkaloid production [5,6,7], as well as that of indole diterpene alkaloids [8,9]. Apart from monocotyledonous plants, some species of Convolvulaceae have also been discovered to contain ergoline alkaloids, leading to the possibility that these dicotyledonous plants might be host plants of fungi in the Clavicipitaceae family [10,11]. Subsequent examination of Convolvulaceae species resulted in the discovery of *Periglandula*, the first clavicipitaceous fungal genus found in dicotyledonous plants [1,12,13].

Previous studies have confirmed that plant-associated fungi are responsible for ergot alkaloid production in Convolvulaceae; both ergot alkaloids and the fungi were eliminated after treating *Ipomoea asarifolia* (Desr.) Roem. & Schult. with fungicides [14]. Moreover, these fungi contain the *dmaW* gene, which is required for the determinant step in ergoline alkaloid biosynthesis [12,15]. Only two species of fungi in the genus *Periglandula* U. Steiner, E. Leistner et Leuchtm. have been reported and named so far, including *Periglandula ipomoeae* U. Steiner, E. Leistner et Schardl from its host plant *Ipomoea asarifolia* and *Periglandula turbinae* U. Steiner, E. Leistner et Schardl from its host plant *Turbina corymbosa* (L.) Raf. [1]. *Periglandula* fungi were found to particularly colonize the adaxial surface of young (unopened) leaves, which are visible to the naked eye as white mycelia spread over the leaf surface [13,16]. These Clavicipitaceae fungi are associated with oil-producing glandular trichomes found on the surface of leaves [1]. It has been suggested that the fungi may uptake volatile oils produced by the glandular trichomes in order to use them for ergot alkaloid biosynthesis [16]. In addition, they appear not to penetrate plant cell walls; however, the fact that the fungi are vertically transmitted via seeds indicates that there is likely some internal growth as well [17]. A clavicipitaceous fungus of grass also inhabits only the external surface of its host plant, but nevertheless is seed-transmissible [18]. The fungus functions as both an epiphyte and an endophyte during different phases of the life cycle of the host plant [19].

There is interest around ergoline alkaloid-producing fungi because of the benefits and properties of ergot alkaloids. These natural products provide the raw materials used for therapeutic drugs of various diseases, including migraines, Parkinson’s disease and uterine hemorrhaging [7,20]. Ergot alkaloids are also known to affect the nervous system, and can, therefore, be used as hallucinogenic drugs, e.g., lysergic acid diethylamide or LSD [7,13]. Considering their pharmacological properties, ergot alkaloids are an important candidate for drug development.

According to previous studies, *Periglandula* species have been discovered to be associated with only the leaves and seeds of Convolvulaceae species [15,16], and other plant parts have not yet been studied. In order to more thoroughly understand the unique seed-transmitted fungus and symbiont–host relationship, this research aims to reveal where and how a species of *Periglandula* interacts with its host plant via molecular, histochemical, anatomical and micromorphological investigation in different plant parts (leaves, stems, roots, flowers, and seeds) of *I. asarifolia*, which is known to be associated with *Periglandula*.

## 2. Materials and Methods

### 2.1. Study Species

*Ipomoea asarifolia* (Desr.) Roem. & Schult belongs to the morning glory family (Convolvulaceae) and is distributed throughout most tropical areas. It is a perennial herb that has leaves with a circular or kidney shape; a cordate base; and a broadly round, emarginated or mucronulate apex. Inflorescences consist of red-purple flowers that have funnel-form corollas [21].

*Ipomoea asarifolia* was collected from the Na Di District, Prachin Buri, Thailand (14°08′ N, 101°44′ E). After confirmation of fungal mycelia on young folded leaf surfaces, the following plant parts were collected from multiple plants for further investigation: young (folded) leaves, mature (opened) leaves, stems, roots, flower buds, mature flowers, young seeds (still green), and mature seeds (completely ripe). The samples were separated and stored as either fresh samples (for stereo microscopy), spirit samples (for scanning electron microscopy, histochemical and anatomical investigation), or frozen samples kept at −20 °C (for molecular analysis). To preliminarily confirm the presence of fungi, young folded leaves of *I. asarifolia* were manually opened and observed using a stereo microscope, before undertaking the methods described below.

### 2.2. Confirmation of Fungal Existence by Molecular Analysis

To confirm that the observed fungus was indeed *P. ipomoeae*, DNA from different plant parts (from multiple plants) were extracted using a CTAB extraction buffer (2% cetyltrimethyl ammonium bromide, 100 mM Tris-HCl pH 8, 25 mM EDTA pH 8, 2 M NaCl, 2% PVP 40, 0.2% 2-mercaptoethanol). Mixed plant-fungal DNA were then processed via PCR to evaluate the presence or absence of the fungus through detection of the *dmaW* gene, which plays a role in ergot alkaloid synthesis within the Clavicipitaceae. The PCR used specific primers to amplify fungal gene sequences, namely, the *dmaWF5* (GACCGTAAACGAGTCAGGAA) and *dmaWR2* (AAATACACCTGGGGCTCG) primers [22,23]. The PCR conditions used were as follows: initial denaturation at 95 °C for 3 min; followed by 35 cycles of 94 °C for 1 min, 55 °C for 1 min, and 72 °C for 1 min; followed by a final extension at 72 °C for 7 min. The PCR products were sequenced by Macrogen Inc. (South Korea) and then compared to sequences in the NCBI database (http://www.ncbi.nlm.nih.gov/; accessed on 20 April 2022) using BLASTN (https://blast.ncbi.nlm.nih.gov/; accessed on 20 April 2022).

### 2.3. Observation of Plant-Fungal Association

Spirit materials from all plant parts were stained with 0.01% fluorescence brightener 28 (calcofluor white) before observation under a BX53 fluorescence microscope (filter: U-FUW Excitation 340–390, emission 420IF). Fungi were detected by the blue fluorescence that results from the binding between polysaccharides in fungal cell walls and fluorescence brightener 28 [12]. This staining method can reveal epiphytic mycelium on the surfaces of plants that are not visible to the naked eye.

Plant-fungal association was also observed using the paraffin method. All plant parts were dehydrated in a tertiary butyl alcohol (TBA) series, followed by immersion in pure TBA with paraffin oil (1:1) and then in pure paraffin oil. The samples were then infiltrated with melted paraplast at 60 °C in the oven, and subsequently embedded in paraffin [24]. The transverse sections were performed using a sliding microtome (Leica SM2000 R) at a thickness of 16 μm. All sections were stained with 0.1% toluidine blue before observation via light microscope.

Scanning electron microscopy (SEM) was performed to provide explicit images of the fungus. Spirit samples were dehydrated through a graded series of ethanol and then critical-point dried in liquid CO_2_ (Hitachi HCP-2). Dried samples were coated with platinum palladium (Hitachi E102 ion sputter) and examined via a field emission scanning electron microscope (Hitachi SU-8010).

## 3. Results

**Figure 1:**
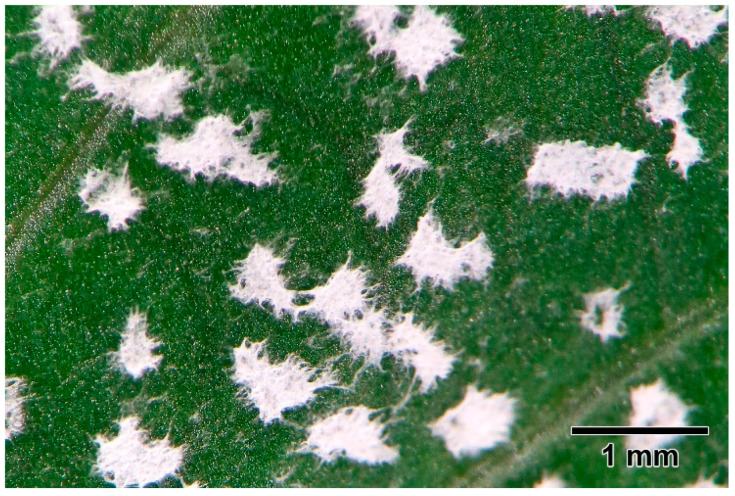
Groups of dense white mycelia on the adaxial surface of a young folded leaf that was manually opened and viewed under stereo microscope.

Stereo visualization of young folded leaves that were manually opened showed that the fungus was distributed only on the adaxial surface, and was also visible to the naked eye. The fungus formed clumps of dense white mycelia packed together (<1 mm in diameter each) and clumps were spread across the entire leaf surface (Figure 1). Apart from the adaxial side of young folded leaves, the fungus was not visible by naked eye on the outer surfaces of other plant parts.

**Figure 2:**
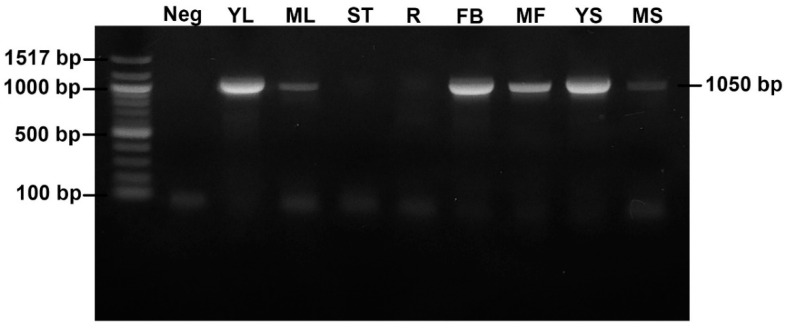
PCR amplification of the dmaW gene in each part of *I. asarifolia.* Neg, negative (water blank); YL, young leaf; ML, mature leaf; ST, stem; R, root; FB, flower bud; MF, mature flower; YS, young seed; MS, mature seed.

To confirm the species of fungus and verify the existence of the fungus in different plant parts, DNA from all parts of *I. asarifolia* were extracted and PCR was performed with fungal-specific primers. The results revealed positive bands with approximately 1050 base pairs in 6 organs (young folded leaves, mature leaves, flower buds, mature flowers, young seeds and mature seeds), but were not observed in the stems or roots (Figure 2). All positive samples showed a 100% match with the fungus *Periglandula ipomoeae* from *I. asarifolia* in the NCBI database (NCBI accession number JN182229.1).

**Figure 3:**
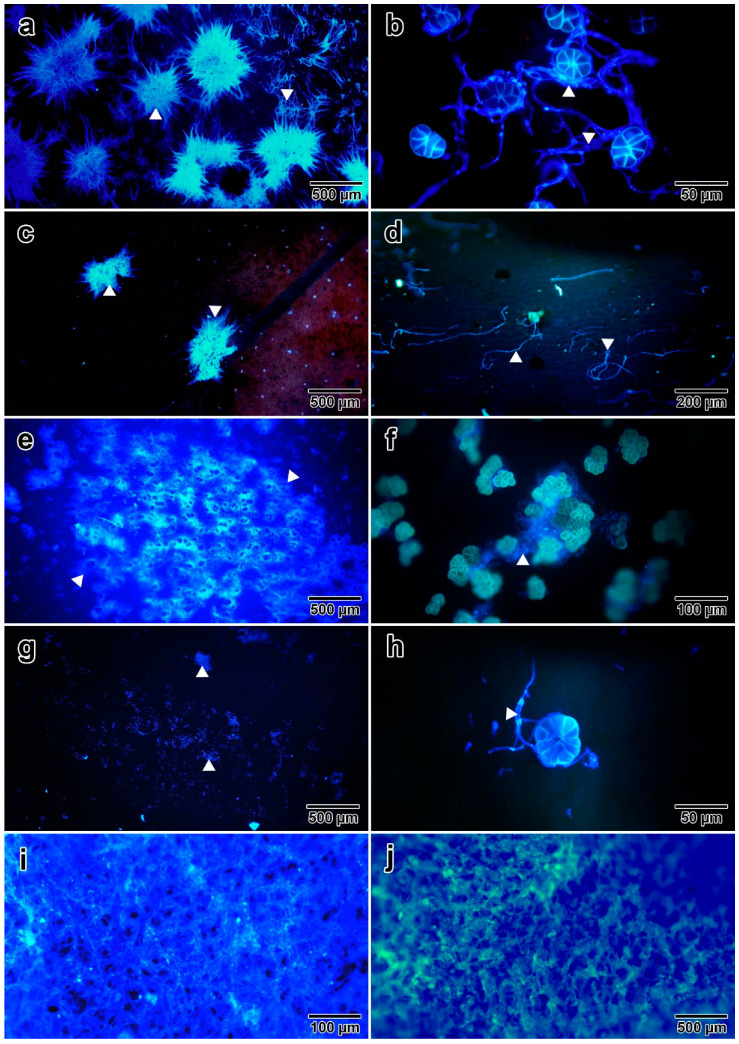
Fungal hyphae (indicated by white arrows) on various surfaces of *I. asarifolia* stained by fluorescence brightener 28: (**a**) adaxial surface of manually opened young leaf; (**b**) interaction between fungal hyphae and glandular trichomes on adaxial surface of young leaf; (**c**) adaxial surface of mature leaf; (**d**) stem; (**e**,**f**) inner side of sepal; (**g**) outer side of sepal; (**h**) outer side of petal; (**i**) surface of young seed; (**j**) surface of mature seed.

Following molecular confirmation of the fungal species, plant–fungal interactions were investigated using fluorescent staining, paraffin sectioning, and SEM. Although microscopic investigation cannot precisely identify the species of fungus, some prominent features aid in distinguishing the fungus and support our interpretations. Fluorescence brightener 28 revealed the presence of mycelia on seven out of eight plant parts (young leaves, mature leaves, stems, flower buds, mature flowers, young seeds and mature seeds). Fungal hyphae appeared to interact with and propagate around the glandular trichomes of *I. asarifolia* in leaves, stems, and flower parts (Figure 3b,d,f,h). The adaxial surface of manually opened young leaves had the highest density of mycelia (>10 large clumps of packed fungal hyphae per 8 × 8 mm of leaf area), consistent with what we observed under stereoscopic observation (Figure 3a). Fluorescence brightener 28 staining also revealed fungal hyphae on the adaxial surface of mature leaves; however, colonies of mycelia were noticeably smaller in size and less dense (fewer than 5 clumps per 8 × 8 mm of leaf area; Figure 3c), compared to the hyphae on young leaves. Meanwhile, the abaxial sides of both young and mature leaves were devoid of the fungus. On the stems, fungal hyphae were not packed together, but were found sparsely distributed along the surface (Figure 3d). Some of the hyphae appeared to interact with glands along the stem, while others did not. Positive staining of fluorescence brightener 28 was also observed on sepals and petals for both flower buds and mature flowers. While no hyphae were observed on the inner sides of petals, numerous groups of hyphae were found on the inner sides of sepals (Figure 3e,f), and a few fungal hyphae were observed on the outer sides of sepals (Figure 3g) and petals (Figure 3h), all of which showed some interaction with glandular trichomes. Additionally, blue fluorescence was found to cover the entire surface of both young (Figure 3i) and mature (Figure 3j) seeds. No hyphae were noticed on the outer surfaces of roots.

**Figure 4:**
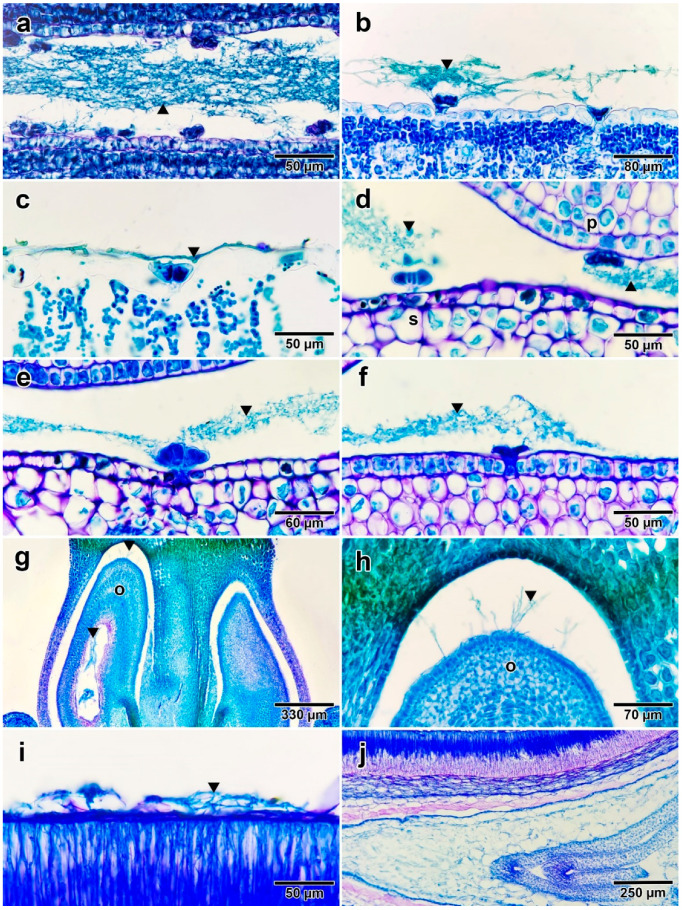
Fungal hyphae (indicated by black arrows) found on different parts of *I. asarifolia*: (**a**) cavity of young folded leaf; (**b**) adaxial surface of young folded leaf that was manually opened; (**c**) adaxial surface of mature leaf; (**d**) cavity between sepal and petal; (**e**) inner side of sepal; (**f**) outer side of petal; (**g**) longitudinal section of ovary; (**h**) close-up of gap between ovule and ovary wall; (**i**) surface of young seed; (**j**) cross section of young seed. Abbreviations: o, ovule; p, petal; s, sepal.

Paraffin sections revealed the presence of fungal hyphae on young leaves, mature leaves, flower buds, mature flowers, and young seeds. Young leaf sections showed clumps of fungal hyphae on the adaxial surface; some clumps interacted with glandular trichomes, as a few hyphae were observed to extend out from the fungal colonies and connect with the glands (Figure 4a,b). Fungal mycelia on mature leaves were difficult to detect, but a few filaments of the fungus could still be observed on the adaxial surface (Figure 4c). There were no differences between the results of flower buds and mature flowers. Transverse sections of flowers revealed several fungal colonies, especially between the inner sides of sepals and outer sides of petals (Figure 4d). Most fungal groups showed association with glandular trichomes on the inner sides of sepals (Figure 4e), while only a few hyphae attached to glands on the outer sides of the petals (Figure 4f). Moreover, fungal hyphae were discovered in floral ovaries, specifically, surrounding the ovules and also occurring inside the ovules (Figure 4g,h). The fungus in young seed sections was difficult to detect, but fungal hyphae-like strands were observed on the seed coat surface (Figure 4i). We could not confirm the presence of fungal hyphae inside of young seeds using anatomical investigation (Figure 4j). Moreover, no fungal hyphae were detected in root or stem sections. We were unable to section mature seeds due to the rigid seed coat.

**Figure 5:**
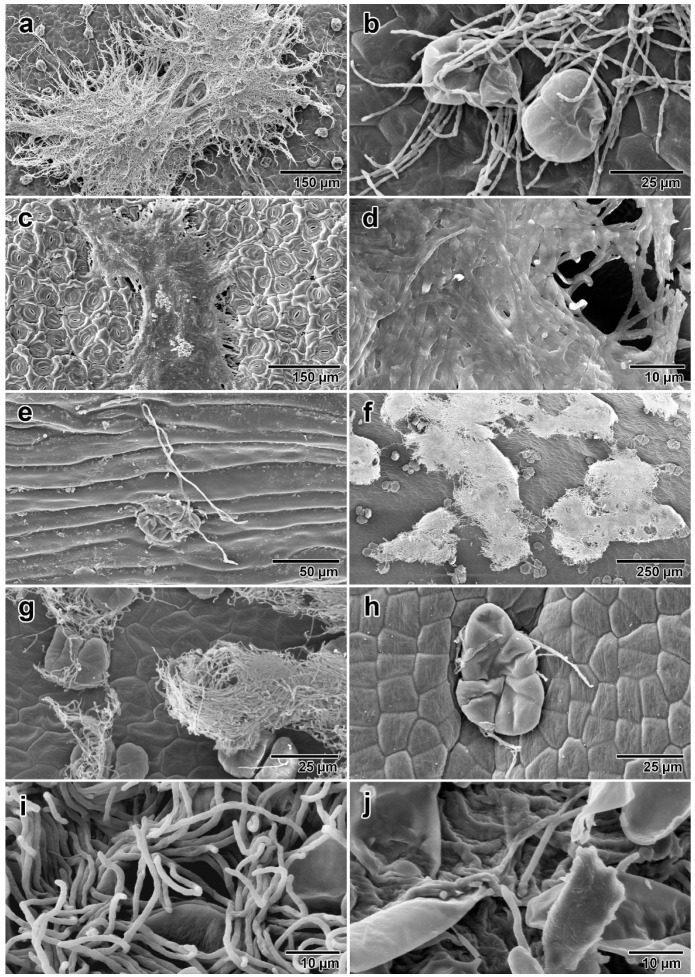
Fungal hyphae from *I. asarifolia* detected by SEM: (**a**) groups of mycelium on adaxial surface of young folded leaf; (**b**) fungal association with glandular trichomes on young leaf surface; (**c**) mycelium on adaxial surface of mature leaf; (**d**) tightly packed hyphae on adaxial surface of mature leaf; (**e**) hypha on surface of stem; (**f**) groups of mycelium on inner side of sepal; (**g**) loosely gathered hyphae associated with glandular trichomes on inner side of sepal; (**h**) hyphae associated with a glandular trichome on outer side of petal; (**i**) numerous hyphae on surface of young seed; (**j**) few hyphae on surface of mature seed.

Finally, SEM revealed clear images of the fungal association with *I. asarifolia*. Corresponding to our histochemical results, SEM revealed the presence of fungal hyphae on young leaves, mature leaves, stems, flower buds, mature flowers, young seeds, and mature seeds (Figure 5). Small hyphae were observed in clumps on the surfaces of young leaves, mature leaves, and sepals (Figure 5a,c,f). However, fungal groups found on mature leaves had a distinctive appearance, as the thick layers of hyphae were so tightly packed that it was difficult to observe individual strands of hyphae (Figure 5d). In contrast, the fungus on young leaves and sepals was more loosely arranged, and numerous separate hyphae were clearly observed (Figure 5b,g). The fungus was observed to be associated with plant glandular trichomes on every part of *I. asarifolia* where glandular trichomes were present, corroborating our histochemical and anatomical results (Figure 5b,e,h). SEM of young seeds disclosed abundant fungal hyphae covering the entire surface of the seeds (Figure 5i), while only a few hyphae were detected on the surface of mature seeds (Figure 5j). No hyphae were found inside of either seed stage. The presence of the fungus in different plant organs is summarized in Table 1.

## 4. Discussion

This study reveals the presence of *Periglandula* fungus in different parts of *I. asarifolia*. After unidentified fungal hyphae were preliminarily observed on young folded leaves by the naked eye and stereo microscope, DNA extraction was performed to confirm the presence of the *Periglandula* species on the plant. Molecular analysis confirmed the existence of *P. ipomoeae* on *I. asarifolia*, and revealed that the fungus is present on the following six organs: young leaves, mature leaves, flower buds, mature flowers, young seeds, and mature seeds. Previous studies have also reported the presence of *P. ipomoeae* on leaves and seeds using molecular investigation, as well as the presence of ergot alkaloids in the leaves and seeds of *I. asarifolia* [12,15], but our study is the first to look for, and find, the *Periglandula* species in the flowers of its host plant.

We also used histochemical and anatomical investigation (fluorescent staining, paraffin sectioning, and SEM) in order to observe how *P. ipomoeae* interacts with its host plant. The three techniques showed similar results, except that fungal hyphae were undetected on the stems and outer sides of sepals when using the paraffin sectioning technique. Histochemistry and SEM revealed that only a few hyphae were found on the stems and outer sides of sepals, which likely explains why we did not detect mycelium on these plant parts via transverse sections. We also found that the fungus formed clumps of packed hyphae on various plant surfaces, especially on the adaxial surfaces of both young and mature leaves, and the inner sides of sepals. The fungus did not penetrate plant cell walls, but did interact with plant glandular trichomes. These findings are similar to those of prior studies that have also reported the presence of *Periglandula* spp. on the young unfolded leaves of *I. asarifolia* and *T. corymbosa* [1,15]. In contrast, other plant parts (e.g., stems and flower petals) seemed to have only a few separate hyphae associated with glandular trichomes on their surfaces. The presence of *P. ipomoeae* on mature leaves was also previously reported [1]; however, there have been no reports of *Periglandula* spp. on plant stems and flowers until now.

In most plant parts that exhibit the fungus, the fungal hyphae showed a connection with plant glandular trichomes. Some evidence indicates that plant trichomes can be the colonization and infection sites of various fungi [25,26]. Association with the oil glands of its host plant is one notable characteristic of *Periglandula* spp., hence the genus name [1,14]. Markert et al. [27] suggested that there must be a transport system between *Periglandula* spp. and their host plants in order to translocate ergot alkaloids from the fungi to the plants. This conclusion came from their findings that they could not detect ergot alkaloids in the fungal mycelium (which have the ergoline alkaloid biosynthesis genes) collected from the adaxial surface of young leaves, whereas they did detect ergot alkaloids in fungus-free leaves of the host plants, which do not have the biosynthesis genes [27]. Given that the *Periglandula* species may use oil from the glands as a precursor for ergot alkaloid biosynthesis [16], the connection between fungal hyphae and plant oil glands may serve as a transportation hub for compound exchange.

In contrast, there were no glandular trichomes on the seed coat surface for the fungus to connect with. Fungal hyphae were detected on surface of seeds in this study. Previous studies have reported the existence of *Periglandula* spp. in the seeds of their host plants by molecular detection [12,15]. The authors of these previous studies proposed that the fungi must grow inside of the seeds, as they can engage in vertical transmission via seeds [12,15]. Nevertheless, we found neither penetration of the fungal hyphae into the seed coat nor presence of hyphae inside the seed. Clay and Schardl [3] studied endophyte symbiosis in grasses and reported that hyphae can asymptomatically invade the seeds and ovules of the host plant and spread after seed germination. Our detection of *P. ipomoeae* on flowers, particularly around and within the ovules, as well as on the seed coat of *I. asarifolia*, provide greater insight into how vertical transmission of *P. ipomoeae* occurs in its host plants.

Our observations from the transverse sections give new insight into how *P. ipomoeae* interacts with its host plants. While most hyphae from the fungal groups are packed together and distributed across plant surfaces, a few hyphae extend out to connect with the plant glandular trichomes. This tenuous connection may be one reason why the fungus tends to gather in less exposed areas, such as in the crevice of young folded leaves and the inner sides of sepals, while only small amounts of mycelium are found on exposed plant parts, such as stems and the outer sides of sepals. With only a few fungal filaments attached to plant glands, they may be easily removed from plant surfaces, such as by rainfall. Prior studies provide support for this conjecture, as rainfall has been shown to significantly reduce fungal conidia of different fungal species (e.g., powdery mildew, *Metarhizium anisopliae*, *Phakopsora pachyrhizi*) on leaf surfaces, and even inhibit subsequent fungal development [28,29,30].

Another factor that affects the presence of mycelia on each plant organ is the density of glandular trichomes. We observed fewer groups of hyphae on mature leaves than on young folded leaves, and we also observed fewer glandular trichomes on mature leaves compared to young leaves (Y. Olaranont, pers. obs.). Similarly, Steiner et al. [31] reported significantly fewer glandular trichomes on expanded (mature) leaves. Moreover, we observed only a few hyphae on the outer sides of petals, which have few glands, and no mycelia on the inner sides of petals, which have no glands at all (Y. Olaranont, pers. obs.). The density of hyphae, number of trichomes per leaf area, and concentration of ergot alkaloids have been found to be positively correlated in both *I. asarifolia* and *T. corymbosa* [31].

In addition to gland density, the developmental stage of the plant part appears to be another factor that affects the amount of mycelium. In this study, we found more fungal mycelia on young plant parts compared to mature parts (with the exception of flowers, where we observed no differences between flower buds and mature flowers). Since many fungal species receive nutrients from their host plants [32,33], it is possible that they reap greater benefits from young organs, where plants typically concentrate resource allocation for growth and development [34,35].

To date, *Periglandula* spp. have mostly been observed on plant surfaces, with no observations of the fungi inside plant tissue. Even for *P. ipomoeae* that we observed inside the ovary, mycelia were only found in the cavity between the ovule and ovary wall, and in the open cavity inside of the ovule itself; no mycelia were observed inside plant tissue. It is difficult to distinguish small fungal hyphae from plant organelles, despite having tried various staining methods [36,37,38]. Thus, we were not able to confirm the presence of the fungus inside seeds by anatomical investigation.

One final noteworthy observation is that there was disagreement between the results of our molecular analysis and the results of histochemical investigation and SEM. Our molecular results confirmed the presence of *P. ipomoeae* in young leaves, mature leaves, flower buds, mature flowers, young seeds and mature seeds. However, histochemical investigation (using fluorescence brightener 28) and SEM revealed that fungal hyphae also colonized the stems of *I. asarifolia*. There are two possible explanations for these conflicting results, which are as follows: (1) the fungus found on stems may be not *Periglandula* spp., therefore, DNA sequencing did not detect them, or (2) the fungus found on stems may be *Periglandula* spp., but the quantity and quality of the fungal DNA were too low to detect by molecular analysis, as only very low densities of fungal hyphae were found on the stems. To answer this question, further investigation is needed, such as using a different method of DNA extraction, ITS-PCR identification, performing fungal culture from hyphae obtained from stems, or testing for the presence of ergot alkaloids in stems.

The results of this study indicate that *P. ipomoeae* colonizes almost every aerial surface of *I. asarifolia*, including reproductive parts, such as flowers and seeds. Our discovery of the fungus on floral organs and within ovules brings us a step closer to understanding this ergot alkaloid-producing fungus and how it interacts with its host plant. However, further investigation is still required to confirm the identity of fungal hyphae found on stem surfaces, to determine how the fungus penetrates inside plant ovules, and to determine whether the fungus inside of the ovules is retained throughout seed development, thus enabling vertical transmission of *P. ipomoeae*. Future studies using *Periglandula*-free plants as a control may help answer some of the current gaps in our knowledge.

## Figures and Tables

**Table 1:** Presence (check mark) or absence (dash) of fungal hyphae observed on different parts of *I. asarifolia* using molecular analysis, fluorescent staining, paraffin sectioning, and SEM. Abbreviations: - = not detected, + = low density of scattered fungal hyphae, ++ = a few small groups of packed hyphae, +++ = many large groups of packed hyphae, NT = not tested.

Plant Parts		Molecular Analysis	Fluorescent Staining	Paraffin Section	SEM
Young folded leaf	Adaxial surface	√	√ (+++)	√	√ (+++)
	Abaxial surface		-	-	-
Mature leaf	Adaxial surface	√	√ (++)	√	√ (++)
	Abaxial surface		-	-	-
Stem		-	√ (+)	-	√ (+)
Root		-	-	-	-
Flower bud and mature flower	Outer side of sepal	√	√ (+)	-	√ (+)
	Inner side of sepal		√ (+++)	√	√ (+++)
	Outer side of petal		√ (+)	√	√ (+)
	Inner side of petal		-	-	-
	Ovules		NT	√	NT
Young seed		√	√ (+++)	√	√ (+++)
Mature seed		√	√ (++)	NT	√ (++)

## References

[1] Steiner U., Leibner S., Schardl C.L., Leuchtmann A., Leistner E. (2011). *Periglandula*, a new fungal genus within the Clavicipitaceae and its association with Convolvulaceae. Mycologia.

[2] Clay K. (1989). Clavicipitaceous endophytes of grasses: Their potential as biocontrol agents. Mycol. Res..

[3] Clay K., Schardl C. (2002). Evolutionary origins and ecological consequences of endophyte symbiosis with grasses. Am. Nat..

[4] Schardl C.L., Young C.A., Moore N., Krom N., Dupont P.Y., Pan J., Florea S., Webb J.S., Jaromczyk J., Jaromczyk J.W., Francis M.M. (2014). Genomes of plant-associated Clavicipitaceae. Advances in Botanical Research.

[5] Porter J.K., Bacon C.W., Robbins J.D., Betowski D. (1981). Ergot alkaloid identification in Clavicipitaceae systemic fungi of pasture grasses. J. Agric. Food Chem..

[6] Torres M.S., Singh A.P., Vorsa N., White J.F. (2008). An analysis of ergot alkaloids in the Clavicipitaceae (Hypocreales, Ascomycota) and ecological implications. Symbiosis.

[7] Florea S., Panaccione D.G., Schardl C.L. (2017). Ergot alkaloids of the family Clavicipitaceae. Phytopathology.

[8] Schardl C.L., Young C.A., Hesse U., Amyotte S.G., Andreeva K., Calie P.J., Fleetwood D.J., Haws D.C., Moore N., Oeser B. (2013). Plant-symbiotic fungi as chemical engineers: Multi-genome analysis of the Clavicipitaceae reveals dynamics of alkaloid loci. PLoS Genet..

[9] Cook D., Lee S.T., Panaccione D.G., Leadmon C.E., Clay K., Gardner D.R. (2019). Biodiversity of Convolvulaceous species that contain ergot alkaloids, indole diterpene alkaloids, and swainsonine. Biochem. Syst. Ecol..

[10] Amor-Prats D., Harborne J.B. (1993). New sources of ergoline alkaloids within the genus *Ipomoea*. Biochem. Syst. Ecol..

[11] Meira M., Silva E.P.D., David J.M., David J.P. (2012). Review of the genus *Ipomoea*: Traditional uses, chemistry and biological activities. Rev. Bras. Farmacogn..

[12] Steiner U., Ahimsa-Müller M.A., Markert A., Kucht S., Groß J., Kauf N., Kuzma M., Zych M., Lamshöft M., Furmanowa M. (2006). Molecular characterization of a seed transmitted clavicipitaceous fungus occurring on dicotyledoneous plants (Convolvulaceae). Planta.

[13] Steiner U., Leistner E. (2012). Ergoline alkaloids in Convolvulaceous host plants originate from epibiotic clavicipitaceous fungi of the genus *Periglandula*. Fungal Ecol..

[14] Kucht S., Groß J., Hussein Y., Grothe T., Keller U., Basar S., König W.A., Steiner U., Leistner E. (2004). Elimination of ergoline alkaloids following treatment of *Ipomoea asarifolia* (Convolvulaceae) with fungicides. Planta.

[15] Ahimsa-Müller M.A., Markert A., Hellwig S., Knoop V., Steiner U., Drewke C., Leistner E. (2007). Clavicipitaceous fungi associated with ergoline alkaloid-containing Convolvulaceae. J. Nat. Prod..

[16] Leistner E., Steiner U., Esser K. (2018). The genus Periglandula and its symbiotum with morning glory plants (Convolvulaceae). The Mycota.

[17] Panaccione D.G., Beaulieu W.T., Cook D. (2014). Bioactive alkaloids in vertically transmitted fungal endophytes. Funct. Ecol..

[18] Clay K. (1994). Hereditary symbiosis in the grass genus *Danthonia*. New Phytol..

[19] Philipson M.N., Christey M.C. (1985). An epiphytic/endophytic fungal associate of *Danthonia spicata* transmitted through the embryo sac. Bot. Gaz..

[20] Watkinson S.C., Boddy L., Money N. (2015). The Fungi.

[21] Staples G.W., Santisuk T., Larsen K. (2010). Convolvulaceae. Flora of Thailand.

[22] Brown A.M. (2013). Detection methods and phylogenetic investigation of the morning glory associated fungal symbiont, *Periglandula*. Master’s Thesis.

[23] Kaur N., Cooper W.R., Duringer J.M., Badillo-Vargas I.E., Esparza-Diaz G., Rashed A., Horton D.R. (2018). Survival and development of potato psyllid (Hemiptera: Triozidae) on Convolvulaceae: Effects of a plant-fungus symbiosis (*Periglandula*). PLoS ONE.

[24] Olaranont Y., Stauffer F., Traiperm P., Staples G.W. (2018). Investigation of the black dots on leaves of *Stictocardia* species (Convolvulaceae) using anatomical and histochemical analyses. Flora.

[25] Łaźniewska J., Macioszek V.K., Kononowicz A.K. (2012). Plant-fungus interface: The role of surface structures in plant resistance and susceptibility to pathogenic fungi. Physiol. Mol. Plant Pathol..

[26] Kim K.W. (2019). Plant trichomes as microbial habitats and infection sites. Eur. J. Plant Pathol..

[27] Markert A., Steffan N., Ploss K., Hellwig S., Steiner U., Drewke C., Li S.M., Boland W., Leistner E. (2008). Biosynthesis and accumulation of ergoline alkaloids in a mutualistic association between *Ipomoea asarifolia* (Convolvulaceae) and a clavicipitalean fungus. Plant Physiol..

[28] Sivapalan A. (1993). Effects of impacting rain drops on the growth and development of powdery mildew fungi. Plant Pathol..

[29] Inyang E.N., McCartney H.A., Oyejola B., Ibrahim L., Archer S.A. (2000). Effect of formulation, application and rain on the persistence of the entomogenous fungus *Metarhizium anisopliae* on oilseed rape. Mycol. Res..

[30] Dufault N.S., Isard S.A., Marois J.J., Wright D.L. (2010). Removal of wet deposited *Phakopsora pachyrhizi* urediniospores from soybean leaves by subsequent rainfall. Plant Dis..

[31] Steiner U., Hellwig S., Ahimsa-Müller M.A., Grundmann N., Li S.M., Drewke C., Leistner E. (2015). The key role of peltate glandular trichomes in symbiota comprising clavicipitaceous fungi of the genus *Periglandula* and their host plants. Toxins.

[32] Rai M., Agarkar G. (2016). Plant–fungal interactions: What triggers the fungi to switch among lifestyles?. Crit. Rev. Microbiol..

[33] Redman R.S., Dunigan D.D., Rodriguez R.J. (2001). Fungal symbiosis from mutualism to parasitism: Who controls the outcome, host or invader?. New Phytol..

[34] Fischer A.M., Gan S. (2007). Nutrient remobilization during leaf senescence. Senescence Processes in Plants.

[35] Chen F.S., Niklas K.J., Liu Y., Fang X.M., Wan S.Z., Wang H. (2015). Nitrogen and phosphorus additions alter nutrient dynamics but not resorption efficiencies of Chinese fir leaves and twigs differing in age. Tree Physiol..

[36] Marques J.P.R., Soares M.K.M., Appezzato-Da-Gloria B. (2013). New staining technique for fungal-infected plant tissues. Turk. J. Bot..

[37] Huang Y.L., Zimmerman N.B., Arnold A.E. (2018). Observations on the early establishment of foliar endophytic fungi in leaf discs and living leaves of a model woody angiosperm, *Populus trichocarpa* (Salicaceae). J. Fungi.

[38] Braga Z.V., dos Santos R.F., Amorim L., Appezzato-da-Glória B. (2019). Histopathology of infection and colonisation of *Elsinoë ampelina* on grapevine leaves. Eur. J. Plant Pathol..

